# Worsening proteinuria and renal function after intravitreal vascular endothelial growth factor blockade for diabetic proliferative retinopathy

**DOI:** 10.1093/ckj/sfaa049

**Published:** 2020-06-28

**Authors:** Michael Shye, Ramy M Hanna, Sapna S Patel, Ngoc Tram-Tran, Jean Hou, Collin Mccannel, Maham Khalid, Mina Hanna, Lama Abdelnour, Ira Kurtz

**Affiliations:** 1 Department of Medicine, Division of Nephrology, UCLA David Geffen School of Medicine, Los Angeles, CA, USA; 2 Department of Medicine, Division of Nephrology, UCI School of Medicine, Irvine, CA, USA; 3 Department of Medicine, Division of Nephrology, Long Beach Memorial Medical Center, Long Beach, CA, USA; 4 Department of Pathology, Division of Renal Pathology, Cedars Sinai Medical Center, Los Angeles, CA, USA; 5 Department of Ophthalmology, UCLA David Geffen School of Medicine, Los Angeles, CA, USA; 6 School of Medicine, University of Queensland-Ochsner Clinical School, Ochsner Health System, New Orleans, LA, USA; 7 Brain Research Center, Los Angeles, CA, USA

**Keywords:** acute kidney injury, aflibercept, bevacizumab, diabetic retinopathy, focal and segmental sclerosis, nephrotic syndrome, proteinuria, ranibizumab, vascular endothelial growth factor, VEGF depletion

## Abstract

Systemic vascular endothelial growth factor (VEGF) inhibitions can induce worsening hypertension, proteinuria and glomerular diseases of various types. These agents can also be used to treat ophthalmic diseases like proliferative diabetic retinopathy, diabetic macular edema, central retinal vein occlusion and age-related macular degeneration. Recently, pharmacokinetic studies confirmed that these agents are absorbed at levels that result in biologically significant suppression of intravascular VEGF levels. There have now been 23 other cases published that describe renal sequela of intravitreal VEGF blockade, and they unsurprisingly mirror known systemic toxicities of VEGF inhibitors. We present three cases where stable levels of proteinuria and chronic kidney disease worsened after initiation of these agents. Two of our three patients were biopsied. The first patient’s biopsy showed diabetic nephropathy and focal and segmental glomerulosclerosis (FSGS) with collapsing features and acute interstitial nephritis (AIN). The second patient’s biopsy showed AIN in a background of diabetic glomerulosclerosis. This is the second patient seen by our group, whose biopsy revealed segmental glomerulosclerosis with collapsing features in the setting of intravitreal VEGF blockade. Though FSGS with collapsing features and AIN are not the typical lesions seen with systemic VEGF blockade, they have been reported as rare case reports previously. In addition to reviewing known elements of intravitreal VEGF toxicity, the cases presented encompass renal pathology data supporting that intravitreal VEGF blockade can result in deleterious systemic and renal pathological disorders.

## INTRODUCTION

There will be an estimated 54 million diabetics in the USA by 2030 [1], and while the prevalence estimates from 2004 are dated, they showed that 4 million adults had diabetic retinopathy (DR) [2]. The development of macrovascular and microvascular complications increases greatly with the duration of time a patient has had diabetes, especially if poorly controlled [3]. Diabetic nephropathy (DN) is diagnosed in 80% of patients with diagnosed DR [4, 5]. There are nearly 125 000 patients receiving vascular endothelial growth factor (VEGF) injections in the USA as of 2015, and a large subset of these patients are vulnerable, and at risk for worsening renal function, proteinuria and end-stage renal disease [6, 7]. DR is due to neo-vascularization caused by VEGF-induced dysregulation of vascular proliferation [8]. It can be remedied by laser photocoagulation and intravitreal VEGF blockade [9].

## VEGF SIGNALING

**FIGURE 1:**
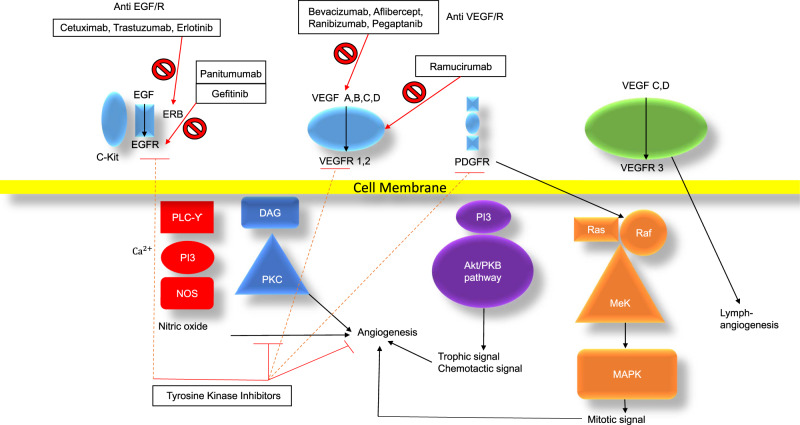
EGFR, VEGF, TKI and mTOR signaling pathways: Akt, protein kinase B (PKB); C-Kit, mast/stem cell growth factor receptor; DAG, diacyl glycerol; ERB, EGFR related-receptor protein (Her2Neu is on type of this); MAPK, mitogen-activated protein kinase; Mek, dual threonine and tyrosine recognition kinase; NOS, nitric oxide synthase; PKC, protein kinase C; PDGFR, platelet-derived growth factor receptor; PI3, phosphatidylinositol-4,5-bisphosphate 3-kinase; PLCϒ, phospholipase C-gamma; RAF, serine/threonine kinase/cellular homolog of viral RAF gene; RAS, rat sarcoma protein; VEGF A–D, vascular endothelial growth factor A–D; VEGFR 1–3, vascular endothelial growth factor receptor 1–3. Adapted from Selamet *et al*. [10].

VEGF inhibitors inhibit a very complex cellular signaling system involved in cell growth, endothelial function and podocyte function [10–12]. The VEGF signaling pathway is linked with the epidermal growth factor receptor (EGFR) pathway at the cellular signaling level. Its downstream mediators are the targets of tyrosine kinase inhibitors (TKIs) [13] and the mammalian target of rapamycin (mTOR) signaling system [14]. Figure 1 demonstrates VEGF signaling and related EGFR signaling, as well as downstream signaling from TKI and mTOR pathways.

## VEGF ANTAGONIST USES IN ONCOLOGIC INDICATIONS

The oncologic uses of VEGF blockade are many, and they are well established as adjunct chemotherapy agents [10–12]. It is equally recognized that they can sometimes have severe systemic side effects [10, 11]. Bevacizumab was the first VEGF blocking agent used systemically in cancer patients. It is a humanized anti-VEGF monoclonal antibody that is currently indicated for non-small cell lung cancer, renal cell carcinoma, breast cancer, colorectal cancer, ovarian cancer, gliomas (a form of brain tumor) and other malignancies [15–21]. Bevacizumab was Food and Drug Administration (FDA) approved for systemic use in 2004 [22].

The evidence is firm that systemic VEGF blockade in oncologic treatment results in worsening hypertension, *de novo* or worsening proteinuria and thrombotic microangiopathy. VEGF blockade can also result in worsening kidney function and irreversible glomerular injury [10–12].

## THE PHYSIOLOGIC ROLE OF VEGF IN THE KIDNEYS AND ENDOTHELIUM

VEGF is an increasingly recognized signal mediator that has been shown to be important in the health of renal podocytes and endothelial cells [11, 12, 23]. Both an excess and deficiency in VEGF signaling have been shown to negatively affect podocyte structure and function. In the podocyte, VEGF signaling is involved in organizing the actin cytoskeleton, including interactions with non-structural Protein 1 and Nephrin [11, 12, 23]. Proper signaling also results in a trophic survival signal through Akt [protein kinase B (PKB)], proper cell cycle function through Ras (Rat-Sarcoma-Protein)/Raf (Serine/threonine-protein kinase) interactions. Nuclear Factor Kappa-light-chain-enhancer of activated B cells-mediated targets of inflammation and renin–angiotensin–aldosterone activation are also suppressed with proper VEGF stimulation [11, 12, 23].

In the endothelium, VEGF signaling is involved in nitric oxide production and vasodilation, a trophic signal for endothelial survival and proper function. Di-Acyl Glycerol-Kinase-Epsilon is also controlled through VEGF signaling, and disruption of this signaling can lead to thrombotic microangiopathy [11, 12, 23]. Hence, significant inhibition of this pathway can easily be shown to lead to podocyte effacement, inflammation and nephrotic syndrome by disruption of the cytoskeleton of the podocyte [11, 12, 23]. There is also a clear link to the thrombotic disorders and hypertension, which would be caused by dysfunction of endothelial cells, clotting dysregulation and nitric oxide synthesis disruption [11, 12, 23].

## OPHTHALMIC USE OF VEGF ANTAGONISTS

The use of VEGF antagonists in ophthalmic diseases was initially administered ‘off-label’, but the US FDA has granted ‘on-label’ indications for aflibercept (Eylea^®^) and ranibizumab (Lucentis^®^) [23]. These agents are indicated for proliferative diabetic retinopathy /diabetic macular edema (DME), central retinal vein occlusion (CRVO) and age-related macular degeneration (AMD) [23].

A typical ophthalmologic regimen would usually be given every month in each eye. The most intensive therapeutic regimens would involve patients getting injections in alternating eyes every 2 weeks with a duration of 1 month between injections in the same eye. The dose of bevacizumab for each injection is 1.25–2.5 mg intravitreally/dose. The typical dose of aflibercept is 2–4 mg given intravitreally/dose. The dose of ranibizumab is 0.3–0.5 mg given intravitreally/dose.

For comparison, the usual systemic dose of bevacizumab is 5–15 mg/kg every 2 weeks. The usual systemic dosage of aflibercept is 2–7 mg/kg every 2 weeks, while ranibizumab is not used systemically. The drug levels with systemic administration are estimated at 100- to 200-fold higher than those achieved with intravitreal injection as cited in FDA package inserts [11, 15–17, 22–26].

## SYSTEMIC ABSORPTION OF INTRAVITREAL VEGF ANTAGONISTS

Pharmacokinetic studies have confirmed that the ophthalmic administration of these agents results in absorption and serum drug levels that are near or greater than the 50% inhibitory concentration (IC50) [26]. The detected serum levels are high enough to result in suppression of more than 50% of intravascular VEGF levels as described by Avery *et al.* [16–18, 27].

The serum level of bevacizumab achieved with intravitreal injection was noted to range from 0.37 nanomoles (nMol) at a minimum and up to 0.77–1.58 nMol at a maximum level. This is greater than the 0.668 nMol IC50. The serum half-life of 18.7 days after three intravitreal injections means that the agent may stay above the IC50 at most for 15–20 days after injection [11, 15–17, 23, 26].

The serum level achieved with intravitreal aflibercept ranges between 0.04 and 0.76 nMol at a maximum level, which is an order of magnitude higher than the IC50 of 0.06–0.07 nMol. The serum half-life is estimated at 11.4 days after 3-month interval injections, and therefore this agent can stay above the IC50 at most for 22–33 days after injection [16, 17, 23, 26].

**FIGURE 2:**
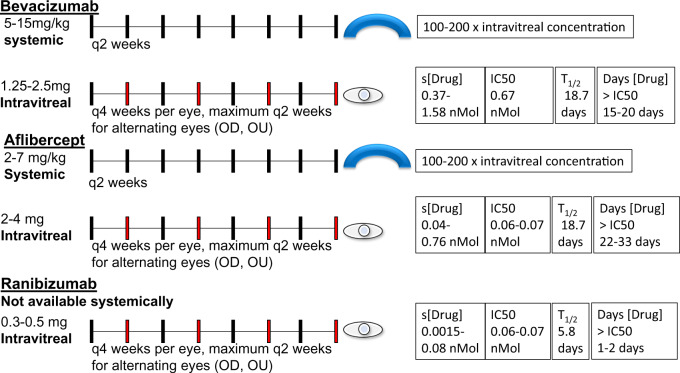
Summary of pharmacokinetic studies showing serum drug levels, serum half-lives from systemically and intravitreally injected VEGF inhibitors. [Drug], concentration of drug every 2 weeks; mg, milligrams; mg/kg, milligram/kilogram; OD, right eye; OU, left eye; q(*x*) weeks, every (*x*) weeks; s[Drug], serum concentration of drug; *T*_1/2_, half-life (in days).

The serum level achieved with intravitreal ranibizumab ranges between 0.0015 nMol and 0.08 nMol; the maximum of this range is near the IC50 of 0.06–0.07 nMol. A serum half-life of 5.8 days was noted, without any evidence of accumulation of drugs between subsequent injections [16, 17, 23, 26]. This agent is only transiently (1–2 days) at higher concentrations than the IC50. Ranibizumab has a shorter half-life in the vitreous humor and is more rapidly cleared because it is a light chain molecule, explaining its lower systemic absorption. Rapid removal of drugs from serum explains the lower half-life, lower systemic concentration, and explains why it has a decreased risk of intravascular VEGF inhibition and resultant systemic effects [16, 17, 23, 26]. See Figure 2 for a depiction of systemic versus intravitreal dosages, serum drug levels as compared with published IC50, half-life and time the drug persists above the IC50.

Several studies corroborate that VEGF suppression is detected by measuring serum VEGF levels [19–21, 28], but the clinical significance is only now being investigated [23]. Accordingly, it is expected that these agents are absorbed at levels capable of causing systemic effects due to the finding of suppressed intravascular VEGF [29]. Bevacizumab and aflibercept are higher potencies, with a longer half-life, stronger absorption have more pronounced VEGF depletion [15–18, 28]. Ranibizumab, on the other hand, tends to have lower absorption and less pronounced VEGF depletion [15–18, 28]. Table 1 depicts the structural and functional differences between VEGF antagonists in common ophthalmologic use.

**Table 1.** VEGF monoclonal antibodies

Agent	Brand name	Weight (kDa)	IC50 (nmol/L)	Binding specificity	Structure
Bevacizumab	Avastin^©^	149	0.66	VEGF-A	IgG1, murine variable region, mAb
Aflibercept	Zaltrap^©^, Eylea^©^	115 (15% glyc)	0.06–0.07	VEGF-A, B	IgG Fc dimerized, dimeric VEGFR 1,2 binding sites
Ranibizumab	Lucentis^©^	48	0.06–0.07	VEGF-A	Kappa light chain mAb fragment

Fc, constant region; g/mol, grams/mole; glyc, glycosylated; IgG, immunoglobulin G; mAb, monoclonal antibody; kDa, kilodalton; mol, mole; mmol/L, millimoles per liter; VEGF-A, B, vascular endothelial growth factor (A, B); VEGFR, Vascular endothelial growth factor receptor.

## SYSTEMIC EFFECTS OF INTRAVITREAL VEGF BLOCKADE

Animal studies have shown that anti-VEGF monoclonal antibody binding can be detected in glomeruli after intravitreal injection. This alters levels of glomerular VEGF and can alter the number of endothelial capillary fenestrations in simian studies [30]. There have been mixed results regarding effects on hypertension [31–34], with one new study linking hypertension and the need for intravitreal VEGF inhibitor use, though the converse relationship cannot be ruled out [35]. Studies have not linked VEGF inhibition to acute kidney injury, but follow-up has been limited [36]. The systemic absorption has known uses in ophthalmology, with the fellow eye effect. This is an observed effect where treating one eye with VEGF blockade improves DR in the contralateral eye [23].

Intravitreal VEGF inhibition raising blood pressure was shown in two studies; one offered an analysis showing 14.3% of patients suffered from a worsening of systolic blood pressure with VEGF blockade [33]. Though no statistically significant change in proteinuria was found in several studies, one recently published study found that 45% of patients with diabetic nephropathy showed increased albuminuria after VEGF blockade was initiated [31]. Another study showed 12.2% of patients with worsening urine albumin/creatinine ratio (UACR) category after intravitreal VEGF blocking antibody injections, but this was not significant [37]. A significant limitation of looking for worsening of categories of proteinuria (as in Glassman *et al.* [37]) is that patients who already have macroalbuminuria (A3 category) would not be captured in the analysis if they experience an exacerbation.

A recent retrospective review of 90 patients, including 45 with diabetic kidney disease, showed worsening proteinuria and renal function in patients with DR and DN over 31 months but was unable to link this progression to greater number of anti-VEGF injections given. Twelve percent of these patients had A3 proteinuria (>300 mg albumin/g protein), as this is the most vulnerable class of patients with DN and DR [38]. This study had significant limitations per the authors because it represented a small, uncontrolled and retrospective study where treatment regimens involved injections in one eye, and systemic absorption was not verified and differences therein were not controlled for [38]. This may indicate that proteinuria and glomerular disease are effects seen within a particular subgroup of patients rather than a side effect seen in all patients receiving these agents [31, 37, 38].

## POPULATION STUDIES AND INTRAVITREAL VEGF BLOCKADE

Some population studies have also raised concerns about all-cause and vascular mortality after initiation of these therapies in patients with AMD [18, 39–41], although these findings were not uniformly observed [42, 43]. The Hanhart studies are worrisome because they consistently showed an increased risk of all-cause mortality, post-cardiovascular (CV) event mortality and post-cerebrovascular (CVA) event mortality. The Hanhart studies were controlled against age- and gender-matched controls. In patients with CVA or CV events, the controls also had a CV/CVA event [39–41]. These events can be plausibly linked with proteinuria, a variable that is not optimally tracked. Table 2 reviews all current studies with data on VEGF absorption, hypertension, renal function and proteinuria.

**Table 2.** Summary of literature on clinical systemic effects of intravitreal anti-VEGF injection

Systemic effect/pathology	*n*	Study type	Reference
A. Evidence of drug absorption and systemic VEGF inhibition			
Absorption in AMD, dec. systemic VEGF (Bev, Aflb) >Ran	56	Prospective observational study	Avery *et al.* [16]
Absorption in AMD/DME/CRVO, dec. systemic VEGF (Bev, Aflib) >Ran	151	Prospective observational study	Avery *et al.* [17]
Absorption of drug in AMD, dec systemic VEGF	610	Retrospective study of RCT data	Rogers *et al.* [20]
Dec. systemic VEGF (Bev, Aflib)	38	Prospective randomized observational study	Zehetner *et al.* [21]
Dec. systemic VEGF (Bev, Aflib)	72	Prospective non randomized clinical study	Hirano *et al.* [28]
Dec. systemic VEGF (Bev, Aflib) >Ran	436	Prospective randomized clinical study	Jampol *et al.* [29]
B. Animal studies showing anti-VEGF binding to glomeruli			
Absorption of drug, binding at glomerulus	N/A	Animal (Simian) study	Tschulakow *et al.* [30]
C. Effects on hypertension after intravitreal injection			
Limited short-term rise in blood pressure at 1 h	135	Prospective observational study	Lee *et al.* [32]
Long- and short-term rise in systolic blood pressure	82	Observational study	Rasier *et al.* [33]
No significant change in blood pressure	57	Observational study	Risimic *et al.* [34]
Higher blood pressure linked to need for more VEGFi	2916	Retrospective study	Shah *et al.* [35]
D. Trial data			
Increased proteinuria 45% of patients (not statistically significant)	40	Prospective observational Study	Bagheri *et al.* [31]
Significant rise in diastolic blood pressure			
Significant rise in hemoglobin and platelets			
No change in eGFR 7–30 days after injection (Bev, Aflib, Ran)	69	Retrospective observational study	Kameda *et al.* [36]
No long-term change in HTN or category of albuminuria	660	Planned retrospective analysis of trial	Glassman *et al.* [37]
No association with # VEGFi injections and proteinuria	43	Retrospective observational study	O’Neill *et al.* [38]
Significant rise in UPCR in patients with preexisting proteinuria	53	Prospective observational study	Chung et.al. [64]
E. Population studies showing increased morbidity and mortality			
Increased risk of CVA in DME patients	N/A	Meta-analysis	Avery *et al.* [18]
Increased AC mortality in AMD patients	1063	Retrospective observational study^a^	Hanhart *et al.* [39]
Increased risk of mortality after MI in AMD patients	211 (with MI)	Retrospective observational study^b^	Hanhart *et al.* [40]
Increased risk of mortality after CVA in AMD patients	948 (with CVA)	Retrospective observational study^b^	Hanhart *et al.* [41]
No finding of CVA, MI, AC mortality in AMD patients	504	Retrospective observational study^b^	Dalvin *et al.* [42]
No finding of increased CVA in DME patients	2541, 690 (with VEGFi)	Retrospective observational study^b^	Starr *et al.* [43]

# , number of (injections); AC, all-cause mortality; Aflib, aflibercept; Bev, bevacizumab; dec., decreased; HTN, hypertension; MI, myocardial infarction; *n*, number of study subjects; Ran, ranibizumab; RCT, randomized controlled trial; VEGFi, vascular endothelial growth factor inhibitors. Green lettering = positive result linking VEGFi and renal outcome; orange lettering = equivocal result; red lettering = negative result.

a Age- and gender-matched control served as comparator group.

b Age- and gender-matched control with a CV or CVA event served as comparator group.

## CLINICAL CASES SHOWING WORSENING RENAL PARAMETERS

At this point, there are multiple published cases reports and series of intravitreal VEGF administration associated with worsening proteinuria, hypertension and glomerular disease [11, 31, 44–54]. We present three additional cases where stable levels of proteinuria and chronic kidney disease (CKD) worsened after initiation of intravitreal VEGF antagonists.

### Clinical cases

#### Case 1

A 58-year-old man with non-insulin-dependent diabetes mellitus (DM) Type 2 diagnosed in 2010, CKD due to diabetic nephropathy, bilateral proliferative DR, bilateral macular edema, hypertension, hyperlipidemia and history of tobacco use was referred by his nephrologist for continued management of CKD Stage 5 and initiation of peritoneal dialysis (PD). He complained of poor appetite without nausea or vomiting. He generally felt fatigued without dyspnea or extremity swelling. No kidney biopsy was performed previously.

The patient was diagnosed with DM upon initial evaluation with his primary care physician in 2010. The initial hemoglobin A1c was 10.8%. His diabetes was well controlled since at least 2013 with a hemoglobin A1c no greater than 6.8%. Oral medications included atorvastatin, calcium acetate, citric acid-sodium citrate solution, diltiazem ER, ergocalciferol, furosemide, hydralazine, sitagliptin, patiromer, pentoxifylline and sevelamer. He had no history of Non Steroidal Anti Inflammatory Drug (NSAID) use, iodinated contrast exposure or ingestion of Chinese herbal medications. There was no history of bisphosphonate administration.

His baseline serum creatinine (Cr) was 3.4–3.8 mg/dL from 2015 to August 2018, and his estimated glomerular filtration rate (eGFR) was 15–20 mL/min. His serum Cr then rose to 5.5 mg/dL in November 2018 and then rose again to serum Cr of 10 mg/dL in February 2019. The eGFR declined correspondingly from 15  to 10 mL/min, and then to 5 mL/min ultimately by November 2018. UACR also increased from a baseline of 1.7–2.7 g albumin/g Cr in April 2018 to 5.2–7.6 g albumin/g Cr in December 2018 to January 2019. The patient’s baseline urine protein/Cr ratio was stable at 2–3.5 mg/dL fromAugust 2015 to July 2018. The urine protein/Cr ratio increased from 2.5 g protein/g Cr in July 2018 to 11.7 g protein/g Cr in January 2019. A renal ultrasound had revealed no structural renal disease. A VEGF-A level was not be obtained on this patient, while he was on intravitreal VEGF blockade therapy, and he is currently off medication. Serum albumin dropped from 4  to 2.6 g/L over the course of the 2018–19 after the initiation of intravitreal anti-VEGF agents.

**FIGURE 3:**
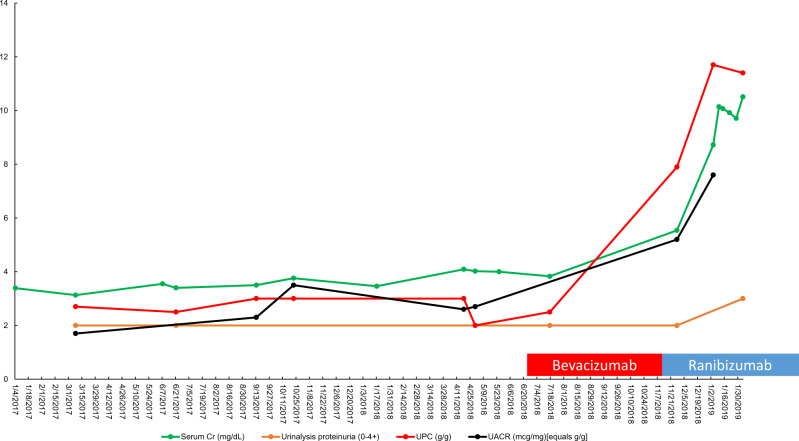
Trend of serum Cr, urinalysis proteinuria, urine protein/Cr ratio and urine albumin/Cr ratio for Patient 1 versus date. Red box, bevacizumab administration; blue box, ranibizumab administration. UPC, urine protein/Cr ratio.

Upon review of records, the patient began following with ophthalmology in May 2018. Due to left vitreous hemorrhage, the patient underwent pars plana vitrectomy, fluid-air exchange and pan-retinal photocoagulation of the left eye in May 2018. He was then initiated on pan-retinal photocoagulation and intravitreal bevacizumab (1.25 mg): 20 June 2018 (L-left eye) and 28 June 2018 (R-right eye). Due to worsening diabetic macular edema, the patient was switched to intravitreal ranibizumab (0.3 mg): 7 December 2018 (R), 2 January 2019 (L), 18 January 2019 (R), 1 February 2019 (L), 1 March 2019 (R), 8 March 2019 (L) and 5 April 2019 (L). These injections together give a total dose of 2.5 mg of bevacizumab and 2.1 mg of ranibizumab. Figure 3 depicts the trends of serum and urine markers of renal function with respect to the timing of initiation of intravitreal VEGF blockade.

His blood pressure remained controlled throughout this time. Despite good control in diabetes and hypertension, the patient had an abrupt worsening in proteinuria and renal function soon after the initiation of intravitreal anti-VEGF agents. The patient declined a renal biopsy, and his serum Cr continued to deteriorate up to 10.5 mg/dL in December 2018. During December 2018 to January 2019, he was transitioned to PD, and he remains on renal replacement therapy (RRT) therapy as of March 2020.

#### Case 2

A 59-year-old male presented to care with a history of poorly controlled DM Type 2 and obesity that was known for 15 years (diagnosed 2003), as well as concomitant hypertension. He had no prior use of NSAIDs, herbal medicines or intravenous iodinated contrast. The patient was taking calcifediol, folic acid, furosemide, lisinopril, amlodipine, sitagliptin, vitamin B6 and vitamin B12. Serum Cr had been stable at 1.1 mg/dL in August 2017, which increased to 2.78 mg/dL by September 2018 following intravitreal VEGF blockade. eGFR was 73 mL/min initially in August 2017 and decreased to 36 mL/min by September–October 2018. In August 2017, the patient had 1.1 g of albuminuria with an increase of in albumin/Cr ratio of 9.4 g albumin/g- Cr in October 2018 over the course of a year. The patient had 1.8 g protein/g Cr in June 2017, which increased profoundly to 16.3 g protein/g Cr by October 2018. Serum albumin also dropped from 4 g/L in August 2017 to 2.7 g/L in May 2019 after intravitreal VEGF blockade was initiated. The patient remained hypertensive throughout, with average blood pressures of 150–170/80–90 mmHg throughout time period of 2018–19 with no change relative to his elevated baseline blood pressure.

Hemoglobin A1c had decreased at the time of presentation with intensive control to 6.6% from a high of 14.3%. Extensive serological testing was mainly negative [human immunodeficiency virus (HIV), hepatitis panel and anti-nuclear antibody (ANA)]. Serum protein electrophoresis (SPEP) did show a faint restricted monoclonal band but with no corresponding monoclonal band seen on immunofixation. Complement levels were normal as well for C3 (144 mg/dL) and C4 (48 mg/dL). Urinalysis showed only trace blood and 3+ proteinuria. Renal ultrasound revealed no hydronephrosis and echogenic kidneys compatible with chronic renal disease. Treatment history was as follows: 1.25 mg of bevacizumab on 16 July 2018 (R); 1.25 mg on 18 July 2018 (L); 1.25 mg on 8 August 2018 (R); 1.25 mg ×2 on 15 September 2018 (R, L); 1.25 mg ×2 on 10 October 2018 (R, L); 1.25 mg on 7 December 2018 (L); 1.25 mg on 15 January 2019 (R); 1.25 mg on 3 March 2019 (L); 1.25 mg on 3 April 2019 (R) and 1.25 mg on 22 May 2019 (R). These injections give a total dose of bevacizumab of 16.25 mg.

Given ongoing deterioration of renal function, a renal biopsy was obtained and showed 15 glomeruli on light microscopy, all without global sclerosis. The glomeruli had diffuse and nodular mesangial matrix expansion with segmental glomerulosclerosis in four glomeruli. One of these lesions of segmental sclerosis demonstrated collapsing features characterized by luminal obliteration with insudates, segmental tuft deflation and associated podocyte hyperplasia with prominent cytoplasmic vacuolization. No crescents or necrotizing features were seen. There was diffuse interstitial edema with a mild to moderate mixed interstitial inflammatory infiltrate, which included both neutrophils and eosinophils. Tubulointerstitial scarring was moderate and arterioles demonstrated afferent and efferent hyalinization. No intravascular fibrin thrombi were seen, nor were there vasculitic changes. Immunofluorescence evaluation demonstrated no significant glomerular staining for immune deposits. Electron microscopy displayed global glomerular basement membrane (GBM) thickening as well as diffuse and nodular mesangial matrix expansion consistent with diabetic glomerulosclerosis. Podocytes display partial foot process effacement. No GBM double contours were noted and endothelial cell fenestrations were intact. The final diagnoses were diffuse and nodular diabetic glomerulosclerosis and segmental glomerulosclerosis with focal collapsing features and interstitial nephritis.

**FIGURE 4:**
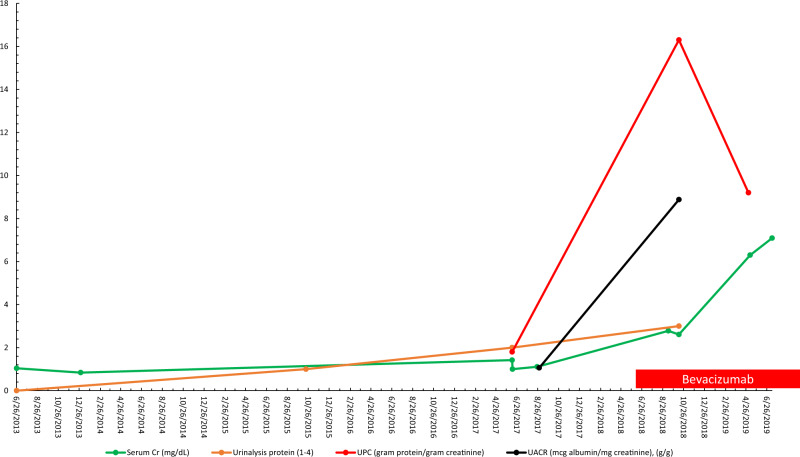
Trend of serum Cr, urinalysis proteinuria, urine protein/Cr ratio and urine albumin/Cr ratio for Patient 2 versus date. UPC, urine protein/Cr ratio.

**FIGURE 5:**
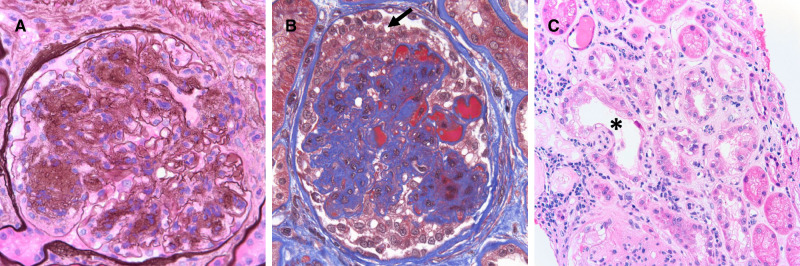
Renal biopsy micrographs for Patient 2 showing diabetic nephropathy and focal and segmental sclerosis with collapsing features. Renal biopsy reveals underlying diffuse and nodular diabetic glomerulosclerosis (**A**, Jones methenamine silver 600×). There were lesions of segmental sclerosis with focal collapsing features (**B**, Trichrome stain 600×) characterized by capillary luminal obliteration by insudates and segmental tuft deflation, with overlying podocyte hyperplasia and prominent cytoplasmic vacuolization (black arrow). There was also concomitant acute tubular necrosis (**C**, asterisk, hematoxylin and eosin, 200×), with interstitial edema and a mixed interstitial inflammatory infiltrate (interstitial nephritis).

The patient’s renal function continued to deteriorate rapidly with the serum Cr increasing to 6.3 mg/dL by May 2019, with eGFR of 11 mL/min, requiring initiation of three times weekly hemodialysis (HD). Figure 4 depicts the trend in serum Cr, urine protein/Cr ratio, UACR and urinalysis proteinuria. Figure 5 shows the renal biopsy findings. A plasma VEGF-A level was not obtained while the patient was on intravitreal VEGF blockade. The patient was transitioned to RRT (HD) as of March 2020.

#### Case 3

A 46-year-old male presented to care with a history of DM Type 2 known for 14 years (diagnosed 2005), requiring both insulin and metformin, but due to deterioration in renal function he was taken off metformin with an urgent referral for a nephrology evaluation. He had no recent use of NSAIDs but had taken naproxen remotely and was told to not take again. He denied the use of herbal medicines or intravenous iodinated contrast. The patient was taking glargine insulin, lisinopril, acetazolamide eye drops, furosemide, baby aspirin and atorvastatin. There was no use of bisphosphonates documented throughout the patient’s medical history.

Serum Cr had been 1–1.2 mg/dL before intravitreal anti-VEGF initiation in June 2018. The serum Cr increased to 2.33 mg/dL by March 2019 after initiation of intravitreal VEGF blockade. eGFR was 46–48 mL/min prior to intravitreal VEGF blockade was initiated in 6/2018 and had been stable, after starting intravitreal VEGF blockade the eGFR had declined to 32 mL/min by 13 March 2019. Unfortunately, there were no baseline levels for UACR and urine protein/Cr ratio (grams protein/gram Cr) that could be located despite a thorough historical search. Albuminuria was measured after starting intravitreal bevacizumab and was found to be at 3.8 g of albumin/g Cr in March 2019 that rose to 4.2 g albumin/g Cr by June 2019. The urine protein/Cr ratio was first checked after VEGF blockade was started and was markedly elevated at 6.35 g protein/g Cr; however, no prior baseline could be found. The serum albumin had dropped from 3.9  to 3.2 g/L over the course of 12 months of intravitreal anti-VEGF therapy from June 2018 to June 2019 . Blood pressure when first seen (after intravitreal VEGF inhibition) was 170s–190s/100s mmHg, higher than the patient’s baseline of 150–170 mmHg prior to June 2018. The patient’s blood pressure was eventually controlled with addition and titration of nifedipine to 140–150 mmHg systolic blood pressure, but with continuing proteinuria and renal dysfunction.

Hemoglobin A1c was controlled at 6.2–6.4%, and extensive serological testing was once again negative (HIV, hepatitis panel, ANA and SPEP). Complement levels were within normal limits. A renal ultrasound showed no hydronephrosis, no masses and increased echogenicity consistent with CKD. Magnetic resonance venography ordered without gadolinium contrast showed no renal vein thrombosis. Urinalysis showed only trace blood and 3+ proteinuria. Treatment history with bevacizumab was as follows: 1.25 mg on 14 June 2018 (R); 1.25 mg on 30 July 2018 (L); 1.25 mg on 24 February 2019 (R) and 1.25 mg ×2 on 26 February 2019 (R, L). He was then switched to ranibizumab 0.3 mg on 25 June 2019 (R, L). These injections give a total dose of bevacizumab of 7.5 mg and a total dose of ranibizumab of 0.6 mg.

**FIGURE 6:**
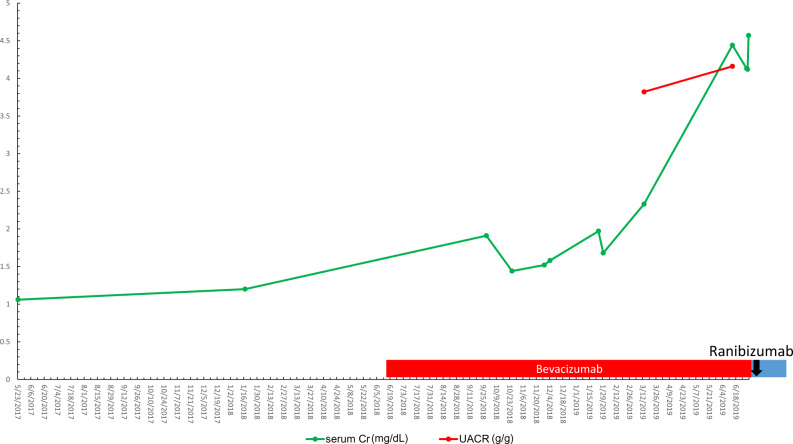
Trend of serum Cr, urinalysis proteinuria, urine protein/Cr ratio and urine albumin/Cr ratio for Patient 3 versus date. Black arrow, ranibizumab initiation; UACR, albumin/Cr ratio.

**FIGURE 7:**
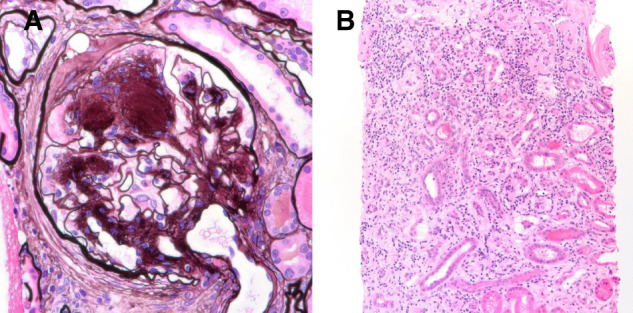
Renal biopsy micrographs for Patient 3 showing diabetic nephropathy and drug-induced acute interstitial nephritis. Renal biopsy revealed diffuse and nodular diabetic glomerulosclerosis (**A**, Jones methenamine silver 600×). There were diffuse interstitial edema and extensive interstitial inflammation (**B**) with associated tubular inflammation and acute necrosis, consistent with interstitial nephritis.

Given the ongoing deterioration of renal function, a renal biopsy was obtained. The light microscopy section showed 33 glomeruli, 12 of which were globally sclerotic (approximately 33% global glomerulosclerosis). Mesangial areas displayed diffuse and nodular expansion by matrix material. No segmental sclerosis or crescents were seen. A patchy and focally dense mixed interstitial inflammatory infiltrate containing few eosinophils was also present. There was severe tubulointerstitial scarring. Immunofluorescence was performed and there was no significant glomerular staining for immune reactants. Electron microscopy revealed global GBM thickening as well as diffuse and nodular mesangial matrix expansion. The final diagnoses were diffuse and nodular diabetic glomerulosclerosis and likely drug/medication-induced interstitial nephritis. The patient’s renal function continued to deteriorate rapidly with a recent serum Cr of 4.57 mg/dL (eGFR of 15 mL/min) by July 2019 and an eventual initiation of HD. Figure 6 depicts the trends in serum Cr, urine protein/Cr ratio, UACR and urinalysis proteinuria. Figure 7 shows the renal biopsy findings. A plasma VEGF level was obtained by June 2019 and found to be low at 36 pg/mL, approximating at the lowest detectable level on this assay (>31 pg/mL). The patient was transitioned to RRT via HD as of March 2020.

## NEW DEVELOPMENTS IN THE INVESTIGATION OF THE SYSTEMIC EFFECTS OF INTRAVITREAL VEGF BLOCKADE

Unrecognized renal and vascular events may be occurring in certain patients treated with VEGF blockade [11, 31, 44–54]. This is especially true in diabetic patients who often have comorbid hypertension, proteinuria and CKD [11, 23]. The concurrence of diabetes and diabetic eye disease with hypertension, proteinuria and CKD means that intravitreal VEGF toxicity may be attributed to underlying comorbidities [10, 11, 23].

It is likely that there are certain factors that may exacerbate the harmful effects of VEGF blockade. Co- or preexisting hypertension, CKD and proteinuria are known to increase the risk of the developing preeclampsia [23]. This process is mediated by soluble fms-like tyrosine kinase 1 resulting in increased VEGF scavenging and signaling depletion [55]. Since preeclampsia is known to be affected by the above renal parameters, it stands to reason that the process of significant VEGF depletion may be inherently dangerous in patients with hypertension, proteinuria and renal disease [23, 55]. It is this subset of patients in whom VEGF depletion may cause worsening hypertension, proteinuria, renal dysfunction and in some instances, glomerulopathies [10–12, 23, 34].

There were previously 23 published case reports of worsening proteinuria, decreased renal function, glomerular diseases and hypertension following initiation of intravitreal VEGF blockade [11, 31, 44–54, 56–59]. There are also three recently described cases of chronic thrombotic microangiopathy (TMA) associated with starting intravitreal VEGF blockade with bevacizumab and aflibercept (under review).

With the addition of these three cases, this brings the total potential published cases to 26, including one instance of focal and segmental glomerulosclerosis (FSGS) with collapsing features (cFSGS) in a patient receiving VEGF blockade for AMD, similar to the biopsy in Case 2. The finding of two such cases is notable, especially given link between cFSGS and TMA noted in literature [51]. This highlights the importance of renal biopsies in identifying unique pathology, which may be induced by intravitreal VEGF blockade.

While systemic VEGF blockade was initially reported to cause TMA, diverse glomerular lesions have been documented, including minimal change disease (MCD), membranous nephropathy and FSGS [10–14, 23]. Notably with intravitreal VEGF blockade, there have been two cases of collapsing FSGS, four published TMA cases and three new ones under review (Table 3).

**Table 3.** Pathology and intravitreal VEGF blockade use

Reference	*n*	Age(s) (years)	Gender	Agent used	Clinical pathology
Hanna *et al.* [11]	4	82, 54, 53 and 65	F	Bev & Ran	Case 1 *de novo* MCD (biopsy+), Cases 2–4 increased proteinuria, CKD progression, HTN worsening
Bagheri *et al*. [31]	18/40	60.3 ± 9.2	33 F and 7 M	Bev	Increased proteinuria in 18/40, 45% of patients
Cheungpasitporn *et al*. [44]	2	52, 67	2 M	Bev	Case 1, MGN and Case 2 TMA (biopsy+)
Diabetic Retinopathy Clinical Research Network [45]	3	NR	NR	Bev	Decreased eGFR
Georgalas *et al*. [46]	2	51 and 68	F and M	Ran & Bev	Decreased eGFR, HD started
Jamrozy-Witkowska *et al*. [47]	1	NR	NR	NR	Decreased eGFR
Kenworth *et al*. [48]	1	88	F	Bev	Increased proteinuria
Khneizer *et al*. [49]	1	74	M	Bev	MGN (biopsy+)
Morales *et al*. [50]	1	56	M	Ran	DN (biopsy+)
Nobakht *et al*. [51]	1	96	F	Bev → Ran → Aflib	cFSGS (biopsy+) + low systemic VEGF level
Pellé *et al*. [52]	1	77	F	Ran	TMA (biopsy+)
Perez-Valdivia *et al*. [53]	1	54	M	Bev	Relapsed MCD (biopsy+)
Sato *et al*. [54]	1	16	F	Bev	Relapsed MCD (biopsy+)
Hanna *et al*. [56]	1	38	F	Bev → Ran	Worsening HTN and proteinuria, lessened with Ran use versus Bev
Touzani *et al*. [57]	1	72	M	Bev	Endotheliosis/possible TMA (biopsy+)
Tran [58]	1	51	M	Bev	AIN (biopsy+)
Yen *et al*. [59]	1	56	M	Bev	TMA (biopsy+)
Hanna *et al*. [manuscript under review]	3	43, 56 and 77	M, F and F	Bev (Cases 1 and 2) Aflib (Case 3)	Cases 1 and 2: DN and chronic TMA (biopsy+) Case 3: FSGS with chronic TMA features (biopsy+)
CCS (Shye *et al*.)	3	46, 58 and 59	3 M	Case 1 Bev → Ran Case 2 Bev Case 3 Bev → Ran	All: increased proteinuria, CKD progression and HD Case 1 Worsening proteinuria, CKD progression and HD Case 2 DN + FSGS with collapsing features + AIN (biopsy+) Case 3 DN + AIN þ low systemic VEGF level (biopsy+)

Biopsy only if (biopsy+) stated. Aflib, aflibercept; AIN, acute interstitial nephritis; Bev, bevacizumab; biopsy+, biopsy obtained; CCS, current case series; F, female; HTN, hypertension; M, male; MGN, membranous glomerulonephritis; *n*, number of patients; NR, not recorded; Ran, ranibizumab.

The coexistence of these different glomerular diseases continues to be noted in association with intravitreal VEGF blockade. Although systemic VEGF and tyrosine kinase blockade should involve different arms of the C-Maf-inducing protein and v-rel avian reticuloendotheliosis viral oncogene homolog A pathways [60], the clinical cases consistently show some overlap with TMA and MCD in both cases of systemic and intravitreal VEGF blockade [10–14, 23].

Overall, these studies suggest an urgent need for closer examination of the pathological effects of long-term VEGF suppression with intravitreal injections [23]. Clinical guidelines are also needed to reduce renal risk in patients where VEGF blockade is needed to maintain acceptable vision [23]. Studies on the use of ranibizumab, with its theoretically improved safety profile, are especially needed [23]. The lack of uniform study results in the literature is an acknowledgment of the complexity of the problem [11, 15–18, 21, 23, 31–34, 36, 37, 39–41, 61]. Despite limited data, risks of intravitreal VEGF inhibition can be approximated to be near 14% for hypertension worsening and 14–45% for proteinuria worsening [31, 33, 37]. The risks are lower than systemic VEGF inhibition administration, where 23.6% of patients have worsening hypertension and 21–63% have worsening proteinuria [62, 63, 64].

There are several gaps in knowledge, the most pressing being the event rate of glomerular disease and proteinuria worsening. The second aspect that needs clarification is which patients tend to absorb VEGF inhibitors intravitreally, and whether there are other modulating factors that need to be considered (disease state). Unique variations in sensitivity to aberrations in VEGF signaling within each patient are also a theoretical point of difference. The variability in systemic exposure of intravitreally injected VEGF inhibitor is another key point of interest [11, 23, 56].

One study that took a step in documenting the observed physiologic changes with VEGF depletion was Bagheri *et al.* [31]; they reported the change in proteinuria as a continuous variable, rather than as KDIGO categories. Interestingly, this study revealed statistically significant changes in diastolic blood pressure similar to the earlier study of Rasier *et al.* [31, 33]. Of note, this study also showed changes in hematological parameters (hemoglobin and platelet count) [31]. This observation is consistent with known endothelial effects of VEGF blockade and can provide additional mechanisms for the pro-thrombotic effects suggested by the studies of Hanhart *et al.* [23, 39–41].

A study that would have the best chance of documenting the systemic effects of intravitreal VEGF inhibition with a reasonable level of confidence would be complex. Kameda *et al.* [36] suggest the need to look at renal function chronically and to assess acute and subacute renal injury markers (besides proteinuria). Glassman *et al.* [37] suggest the need to look at proteinuria as a continuous variable, rather than as a categorical measure. Bagheri et al. [31] suggest the need to confirm VEGF inhibition, and Avery et al. [16, 17] suggest the need to measure drug levels after intravitreal injection. O’Neill *et al.* [38] suggest the need to look at the changes in proteinuria prospectively. Given these considerations, a well-designed study to adequately assess the systemic effects of intravitreal VEGF inhibition would need to be well controlled. It also must be well powered, especially if the event rate is low or the effects are mostly detected in a subgroup of patients.
